# Determining the Degree of Promiscuity of Extensively Assayed Compounds

**DOI:** 10.1371/journal.pone.0153873

**Published:** 2016-04-15

**Authors:** Swarit Jasial, Ye Hu, Jürgen Bajorath

**Affiliations:** Department of Life Science Informatics, B-IT, LIMES Program Unit Chemical Biology and Medicinal Chemistry, Rheinische Friedrich-Wilhelms-Universität, Bonn, Germany; Kyushu University, JAPAN

## Abstract

In the context of polypharmacology, an emerging concept in drug discovery, promiscuity is rationalized as the ability of compounds to specifically interact with multiple targets. Promiscuity of drugs and bioactive compounds has thus far been analyzed computationally on the basis of activity annotations, without taking assay frequencies or inactivity records into account. Most recent estimates have indicated that bioactive compounds interact on average with only one to two targets, whereas drugs interact with six or more. In this study, we have further extended promiscuity analysis by identifying the most extensively assayed public domain compounds and systematically determining their promiscuity. These compounds were tested in hundreds of assays against hundreds of targets. In our analysis, assay promiscuity was distinguished from target promiscuity and separately analyzed for primary and confirmatory assays. Differences between the degree of assay and target promiscuity were surprisingly small and average and median degrees of target promiscuity of 2.6 to 3.4 and 2.0 were determined, respectively. Thus, target promiscuity remained at a low level even for most extensively tested active compounds. These findings provide further evidence that bioactive compounds are less promiscuous than drugs and have implications for pharmaceutical research. In addition to a possible explanation that drugs are more extensively tested for additional targets, the results would also support a “promiscuity enrichment model” according to which promiscuous compounds might be preferentially selected for therapeutic efficacy during clinical evaluation to ultimately become drugs.

## Introduction

Polypharmacology is an emerging theme in pharmaceutical research [1–3]. It refers to increasing evidence that the therapeutic efficacy of many drugs depends on multi-target engagement. For example, this is by now well established for protein kinase inhibitors used in cancer therapy [4]. In the context of polypharmacology, compound promiscuity has been defined as the ability of small molecules to specifically interact with multiple targets [5,6], as opposed to engaging in non-specific or apparent interactions. Accordingly, so-defined promiscuity should not be confused with undesired pan-assay interference (PAINS) [7] or aggregator characteristic of compounds, giving rise to many false-positive assay readouts and doomed compound optimization efforts. PAINS are typically reactive under assay conditions and the different types of undesired reactions associated with major classes of PAINS have been detailed [8]. Rather, promiscuity can be rationalized as the molecular basis of polypharmacology, which might also result in unwanted side effects due to specific target engagement.

Given the increasing sizes of compound databases and volumes of activity data, promiscuity of drugs and bioactive compounds can be estimated through computational data mining. Several studies have attempted to determine the numbers of targets drugs or bioactive compounds are known to be active against, focusing on premier public domain databases such as DrugBank [9], a major source of drug-target annotations, ChEMBL [10,11], the major public repository of compound activity data from medicinal chemistry, or the PubChem BioAssay collection [12], the major public repository of screening data, as well as various commercial compound databases. For example, surveys of drug targets have indicated that drugs interact on average with two to seven targets, depending on their primary target families and therapeutic areas, and that more than 50% of current drugs might interact with more than five targets [3]. On the basis of most recent estimates focusing on high-confidence activity data (i.e., well-defined single-target assays and precise activity measurements), approved drugs bind on average to 5.9 targets, whereas bioactive compounds from medicinal chemistry sources bind to 1.5 targets [13]. Interestingly, the average degree of compound promiscuity (i.e., average number of targets a compound is active against) was not notably higher for compounds active against major therapeutic targets such as G protein coupled receptors (GPCRs) or protein kinases [13]. Furthermore, mean degrees of promiscuity were not significantly higher for active compounds from confirmatory assays with, on average, 2.5 targets per compound [13,14]. Moreover, the degree of promiscuity of bioactive compounds covering the current spectrum of therapeutic targets did not significantly increase over time when high-confidence activity data were analyzed, despite the rapid growth in assay and activity data during recent years. For example, between 2004 and 2014, when most significant data growth occurred, detectable compound promiscuity remained essentially constant, with on average 1.5 targets per bioactive compound [15]. When promiscuity of drugs was followed over time, moderate increases in the degree of promiscuity were detected, albeit larger than for bioactive compounds, with the average degree increasing from 1.5 in 2000 to 3.2 in 2014 [16]. It was also observed that average degrees of promiscuity of drugs were frequently influenced by small numbers of highly promiscuous drug molecules [13]. Taken together, these studies have indicated that drugs are on average much more promiscuous than bioactive compounds, which are overall characterized by relatively low degrees of detectable promiscuity [13,15,16], especially on the basis of high-confidence activity data.

Considering the very large amounts of compound activity data that are already available [17,18], data mining should be expected to yield statistically meaningful promiscuity estimates [18]. On the other hand, there is the frequently discussed issue of data incompleteness [19], referring to the fact that not all available compounds have been tested against all targets. The generation of a complete compound-target activity matrix has been put forward as the ultimate goal of chemogenomics [20], which will most likely remain elusive. Regardless, due to data sparseness, the detectable degree of compound promiscuity might often be lower than true promiscuity, although it is unclear how large discrepancies might be.

In this context, it must also be taken into consideration that major compound repositories such as ChEMBL and DrugBank, upon which promiscuity estimates are based, collect activity annotations of compounds reported in the literature, but do not contain assay frequency or inactivity information, which is typically not reported. No major public compound database contains information of how many times a compound might have been tested so far against how many targets. Therefore, it is not possible, for example, to relate promiscuity degrees to assay frequency across different targets.

One possibility to extend promiscuity analysis through inclusion of assay frequency information is provided by screening data available in the public domain, with PubChem being the major repository. While it is not possible to directly access assay frequency information on a per compound basis, the data are available and it can be determined how many times a compound was tested in different screening assays and how often -and against which targets- it was found to be active. Recently, a web-based search tool has been introduced to retrieve such information from PubChem for individual query compounds [21]. However, for global and large-scale promiscuity analysis, assay and activity profiles must be determined systematically for all source compounds and analyzed in context.

In light of the above, we have reasoned that computational compound promiscuity analysis might be brought up to the next level by examining activity profiles of compounds that have been extensively assayed, thus addressing data sparseness issues in a previously unconsidered manner. To these ends, we have undertaken a large-magnitude analysis on the basis of currently available PubChem assay data. In a first data curation step, it was determined for each screening compound how often it was assayed and found to be active in primary screens as well as confirmatory assays. In the second step, promiscuity analysis was carried out for a large number of extensively tested compounds. In the following, our analysis and the results are presented in detail.

## Material and Methods

### Assay Categories

Assay data were taken from the PubChem BioAssay collection (accessed on 7^th^ September 2015) [12], which contains different categories of assays including primary and confirmatory assays. Primary assays represent original screening data in which the activity assessment is based on percentage inhibition from a single dose. In this case, a compound is classified as active if it reduces target activity below an assay-specific threshold of residual activity. The threshold is often determined on the basis of the activity value distributions resulting from the screen. Accordingly, primary screens produce activity annotations of test compounds (i.e., active vs. inactive) but often not activity values. By contrast, confirmatory assays monitor activity measurements at varying compound concentrations and typically yield IC_50_ values derived from titration curves. In biological screening, it is common practice to re-evaluate initial screening hits in confirmatory assays. However, not all primary assays in PubChem have confirmatory counterparts and vice versa, for at least two reasons. First, primary or confirmatory assays are often independently deposited; second, increasing numbers of initial screens also use varying concentrations of test compounds for activity measurements and are thus confirmatory in nature. In general, activity annotations from primary screens have lower confidence than activity values from confirmatory assays, suggesting to best analyze them separately.

### Data Collection

Primary and confirmatory assays were selected, as described below. From all available primary assays, only RNA interference (RNAi) screens were removed. Accordingly, all chemical screens were retained including primary cell-based assays for which no individual target was specified. For confirmatory assays, a series of selection criteria was applied using the PubChem BioAssay search interface [22]. First, “*On Hold BioAssays*” was set to “no hold”. Second, the type of bioassays was specified by setting “*Substance type*” to “chemical”; “*Screening stage*” to “confirmatory, dose-response”; and “*Target*” to “single”. Third, the “*Target type*” was set to “protein target”. Accordingly, all confirmatory assays in which chemical compounds were tested against single target proteins with dose-response measurements were selected. Fourth, “*Activity (IC*_*50*_, *etc)*” was set to “specified” and “*Activity outcome*” to “active”.

From each qualifying primary or confirmatory assay, only compounds classified as active or inactive were taken, whereas compounds with designations such as unspecified or inconclusive were discarded. For promiscuity analysis, compounds were prioritized that were tested in both primary and confirmatory assays, as rationalized below. For each compound, its identifier in PubChem (i.e., PubChem cid), the number of primary and confirmatory assays it was tested in, the number of primary and confirmatory assays in which it was active, and the number of unique targets from primary and confirmatory assays with activity were recorded.

The complete set of 437,257 compounds with assay and activity information has been made freely available as a ZENODO deposition [23].

### Assay vs. Target Promiscuity

In our analysis, two types of promiscuity were distinguished. The degree of assay promiscuity was defined as the number of assays in which a compound was active. Assay promiscuity was determined by collecting all activity annotations from primary and confirmatory assays, respectively. Hence, different assays for the same target were counted individually. In addition, the degree of target promiscuity was defined as the number of unique targets a compound was active against across all assays. As a hypothetical example, a compound C was tested in assays 1–5 for a target T_1_ and in assays 6–10 for another target T_2_ and found to be active in assays 1, 2, 3, 8, and 10. Then, the corresponding assay and target promiscuity for C was five and two, respectively, indicating that the compound was active in a total of five assays against two targets. If another compound would be tested in 50 assays and found to be active in, for example, 14 against the same two targets, its assay promiscuity would be 14 and its target promiscuity would still be two. Hence, this would further differentiate between compounds having the same degree of target promiscuity. Therefore, these two promiscuity measures are complementary in nature. If no large and/or systematic discrepancies between assay and target promiscuity would be observed, there would be no indication of potential assay bias or false negatives that might affect target promiscuity analysis. Hence, considering assay and target promiscuity in context provides additional information. We also note that the degree of assay promiscuity of a compound may exceed its degree of target promiscuity, whereas target promiscuity cannot exceed assay promiscuity. Assay and target promiscuity were separately determined for compounds from primary and confirmatory PubChem assays.

## Results

### Assay and Compound Selection Strategy

A total of 1358 qualifying primary and 1823 confirmatory assays were obtained. Primary assays included 297 cell-based assays from which only assay promiscuity but not target promiscuity was determined. From primary and confirmatory assays, 836,585 and 457,842 unique compounds were selected, respectively, as reported in Table 1. These assays were directed against 476 (primary assays) and 632 (confirmatory) targets. Taken together, these assays covered a total of 824 unique targets. Furthermore, from all assays, a total of 146,270,306 and 37,808,671 assay-compound records were assembled, each of which reported the activity or inactivity of a given compound in an individual assay (Table 1).

**Table 1:** Assay, target, and compound statistics.

Number of		Primary	Confirmatory
**Assays**		1358	1823
**Targets**		476	632
**Compounds**		836,585	457,842
	**All**	146,270,306	37,808,671
**Assay-compound records**	**Activity**	1,313,226	611,968
	**Inactivity**	144,957,080	37,196,703

From the PubChem BioAssay collection, the number of qualifying primary and confirmatory assays and corresponding targets is reported. In addition, the number of unique compounds tested in these assays is given. Furthermore, the total number of assay-compound records including active and inactive compounds is provided.

Next, the two large sets of compounds from primary or confirmatory assays were further compared. A subset of 437,257 compounds was tested in both primary and confirmatory assays. The remaining 399,328 and 20,585 compounds were evaluated only in primary or confirmatory assays, respectively. Of nearly 400,000 compounds tested exclusively in primary assays, ~73% were only evaluated in one to 10 primary assays. By contrast, only 1.5% of these compounds were tested in more than 50 assays. Furthermore, nearly 91% of these compounds were found to be consistently inactive in all primary assays they were tested in. These findings indicated that compounds tested exclusively in primary assays had low assay frequency and were predominantly inactive and thus not suitable for our promiscuity analysis. Similarly, ~75% of the 20,585 compounds exclusively tested in confirmatory assays were only evaluated in one to 10 and only ~4% of these compounds were tested in more than 50 assays. Hence, these infrequently assayed compounds were also not considered suitable for promiscuity analysis.

By contrast, the 437,257 compounds that were tested in both primary and confirmatory assays exhibited distinctly different assay frequencies. In this case, ~95% of the compounds were tested in more than 50 primary and/or confirmatory assays. Moreover, ~85% of these compounds were evaluated in a total of more than 100 assays. Hence, this subset of 437,257 compounds was extensively tested in both assay categories and strongly preferred for our analysis.

### Assay Frequency Distribution

**Fig 1:**
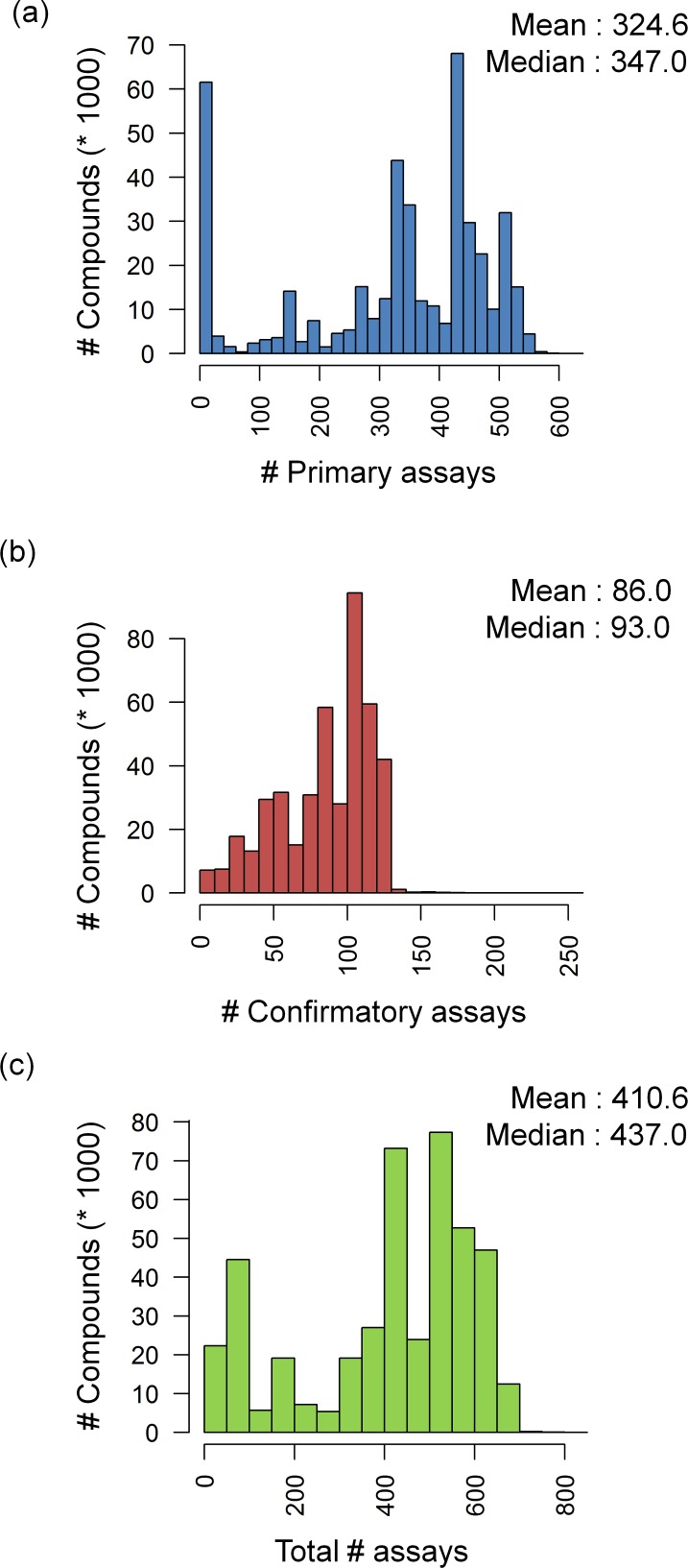
Assay frequency. Reported is the distribution of compounds tested in increasing numbers of (**a**) primary and (**b**) confirmatory assays. In (**c**), both assay categories are combined. In each case, the mean and median number of assays in which a compound was tested is provided.

Fig 1 reports assay frequencies for the 437,257 compounds in detail. In Fig 1A and 1B, the distribution of compounds over primary and confirmatory assays is shown, respectively. The majority of these compounds were tested in hundreds of primary assays, with a mean of 325 assays per compound and a median of 347 assays. In addition, many compounds were also evaluated in more than 100 confirmatory assays (with a mean of 86 and median of 93 assays per compound). Fig 1C shows the distribution for combined primary and confirmatory assays, which confirms that most compounds were extensively evaluated, with a mean of 411 assays per compound and a median of 437 assays. More than 287,000 compounds were tested in a total of 400–848 assays. Hence, the selected compounds provided an unprecedented source for promiscuity analysis.

### Consistently Inactive Compounds

**Fig 2:**
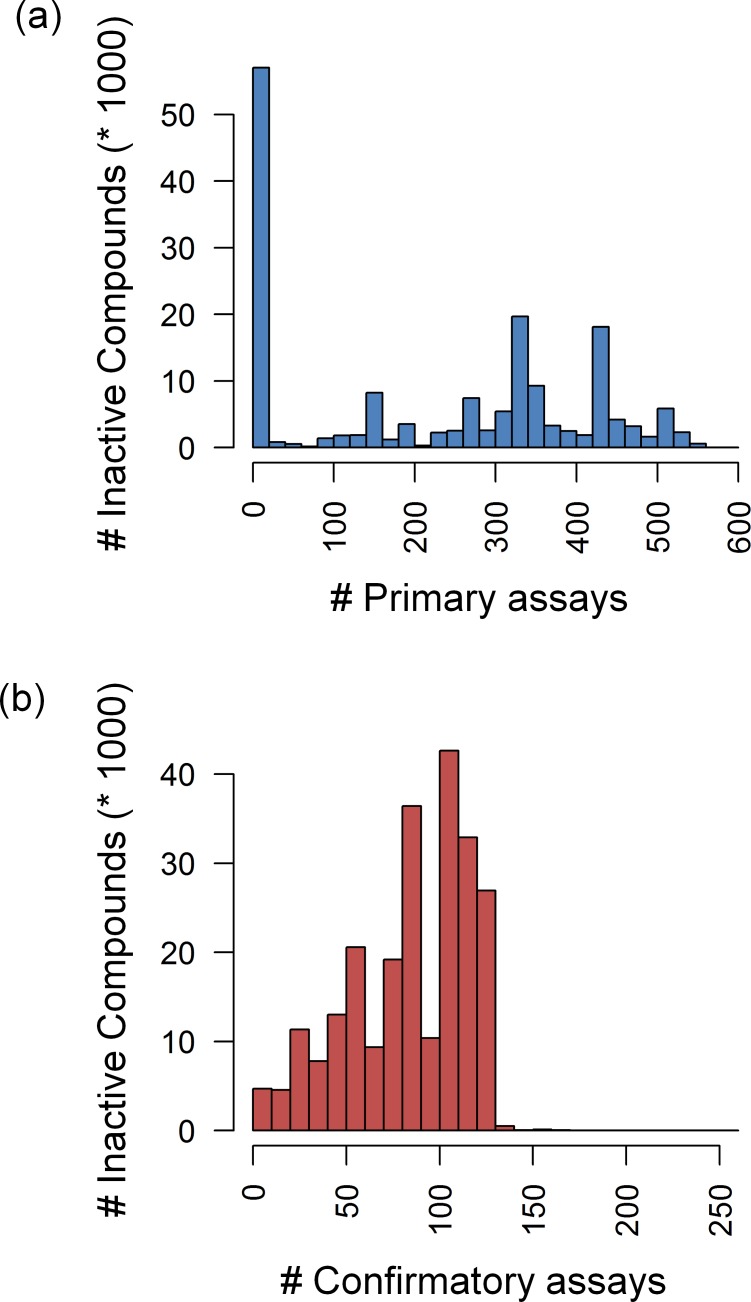
Inactive compounds. Reported is the distribution of compounds that were consistently inactive in increasing numbers of (**a**) primary and (**b**) confirmatory assays.

**Fig 3:**
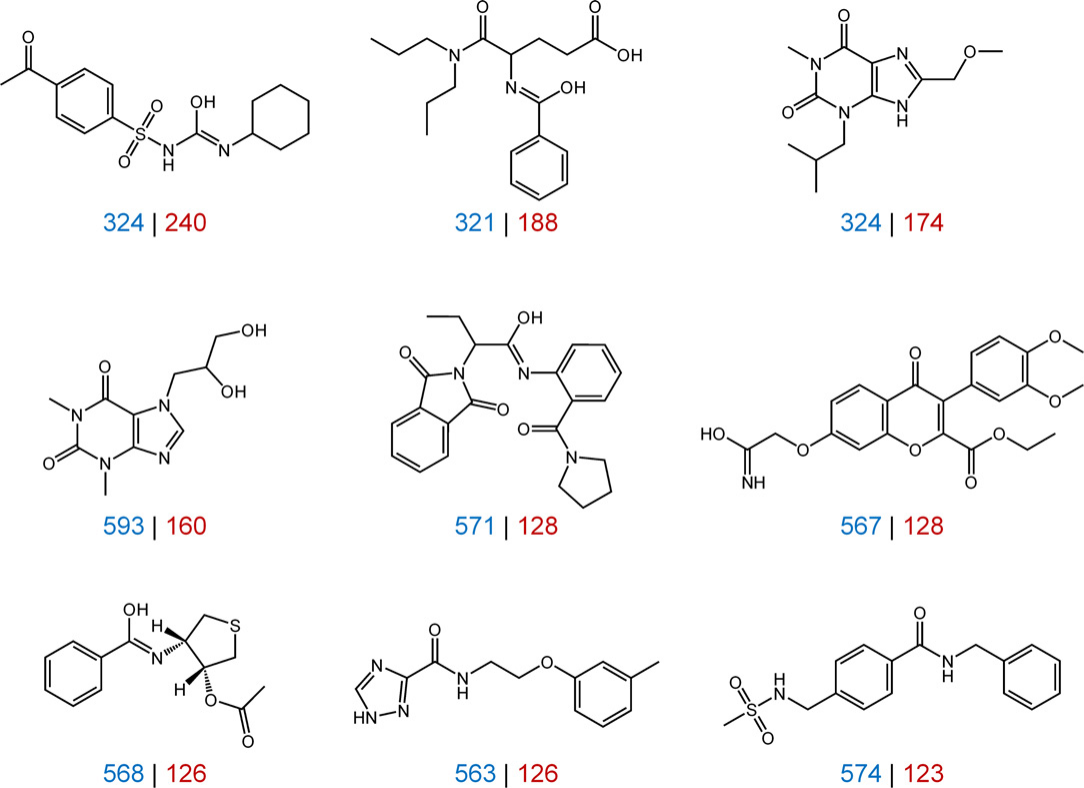
Exemplary inactive compounds. Shown are nine compounds that were consistently inactive in all assays. For each compound, the number of primary and confirmatory assays it was tested in is reported in blue and red, respectively.

Although the compounds were tested in hundreds of assays against hundreds of targets, large numbers of consistently inactive compounds were detected, as reported in Fig 2. In primary (Fig 2A) and confirmatory assays (Fig 2B), a total of 169,839 and 240,650 compounds were consistently inactive, respectively. Furthermore, 119,256 compounds were found to be consistently inactive in both primary and confirmatory assays. Fig 3 shows examples of structurally diverse compounds that were extensively tested, often in nearly or more than 700 assays, yet consistently inactive. The observation that 27.3% of the subset of extensively tested compounds was not active in any assay also indicated that there was no general tendency to produce false-positive assay signals, despite very large number of assays that were considered. Furthermore, these findings might also be viewed in light of recently described “dark chemical matter”, i.e., compounds that have been identified as consistently inactive in high-throughput screening assays of drug discovery projects but that might nonetheless have interesting activities and functional effects in other assay formats [24].

### Compound Promiscuity

**Fig 4:**
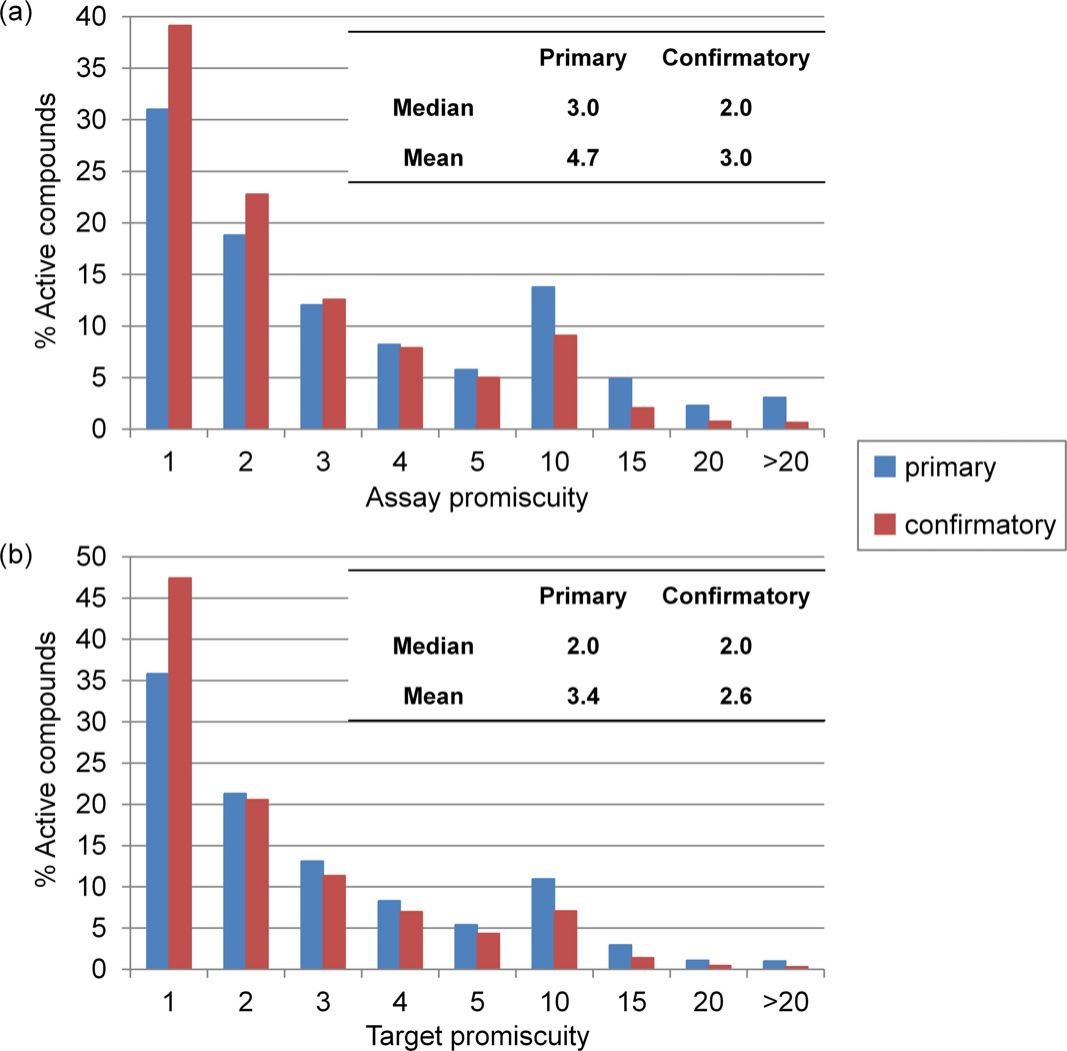
Assay and target promiscuity. Reported are the percentages of compounds with increasing degrees of (**a**) assay and (**b**) target promiscuity. In addition, average and median degrees of assay and target promiscuity are reported.

**Fig 5:**
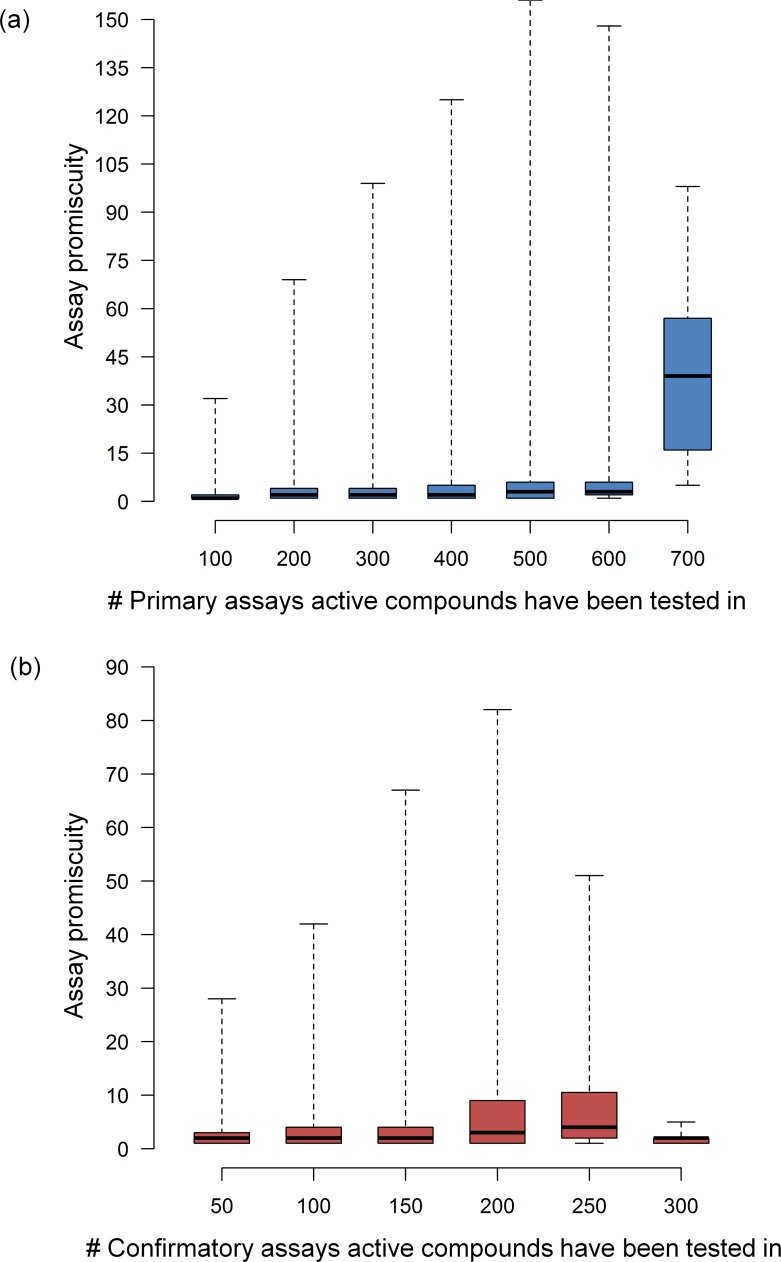
Assay frequency vs. assay promiscuity. For increasing numbers of (**a**) primary and (**b**) confirmatory assays, the distribution of assay promiscuity is reported in a box plot format. The plot gives the smallest degree of assay promiscuity (bottom line), first quartile (lower boundary of the box), median value (thick line), third quartile (upper boundary of the box), and largest degree of assay promiscuity (top line).

**Fig 6:**
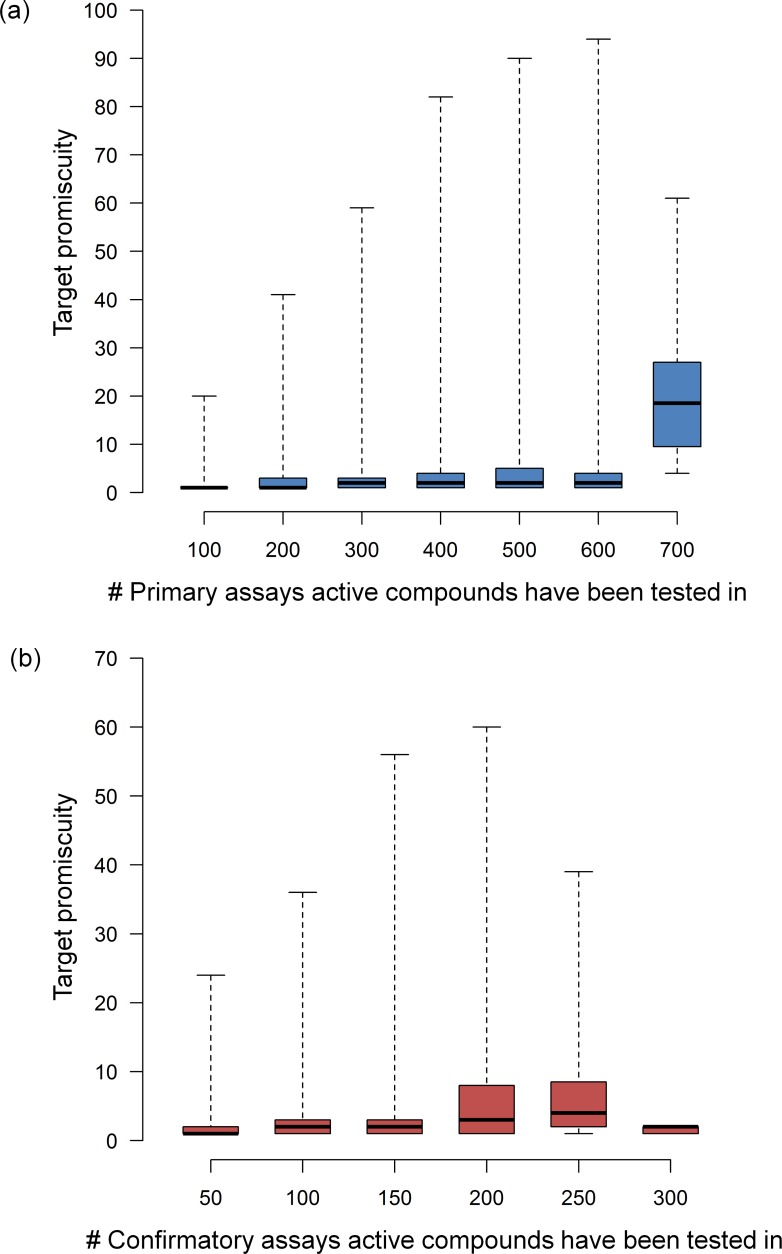
Assay frequency vs. target promiscuity. For increasing numbers of (**a**) primary and (**b**) confirmatory assays, the distribution of target promiscuity is reported in box plots according to Fig 5.

As the primary focal point of our analysis, we then systematically determined assay and target promiscuity for all active test compounds including 267,418 and 196,607 compounds from primary and confirmatory assays, respectively. Fig 4 shows the distribution of compounds that were active in increasing numbers of primary or confirmatory assays. In Fig 4A, assay promiscuity is monitored. On average, a compound was active in 4.7 primary and 3.0 confirmatory assays, with median values of 3.0 and 2.0, respectively. These values were lower than we anticipated. As shown in Fig 4B, and as expected, target promiscuity was lower than assay promiscuity. The average degree of target promiscuity in primary and confirmatory assays was 3.4 and 2.6, respectively, with a median degree of 2.0 in both cases. The observation that mean values were generally slightly or moderately higher than medians was attributed to the presence of a small proportion of highly promiscuous compounds, as further discussed below. Fig 5 reports changes in the degree of assay promiscuity for compounds tested in increasing numbers of primary (Fig 5A) and confirmatory assays (Fig 5B). In primary assays, median assay promiscuity essentially remained constant over increasing numbers of assays, except for a statistically small sample of compounds tested in 600 to 700 assays where an increase was noted. Similar observations were made for confirmatory assays, with the exception of a moderate increase in the spread of promiscuity degrees for compounds tested in 150–250 assays. Fig 6 monitors changes in the degree of target promiscuity for compounds evaluated in increasing numbers of primary (Fig 6A) and confirmatory assays (Fig 6B). The distributions and median degrees of target promiscuity closely corresponded to those of assay promiscuity.

**Fig 7:**
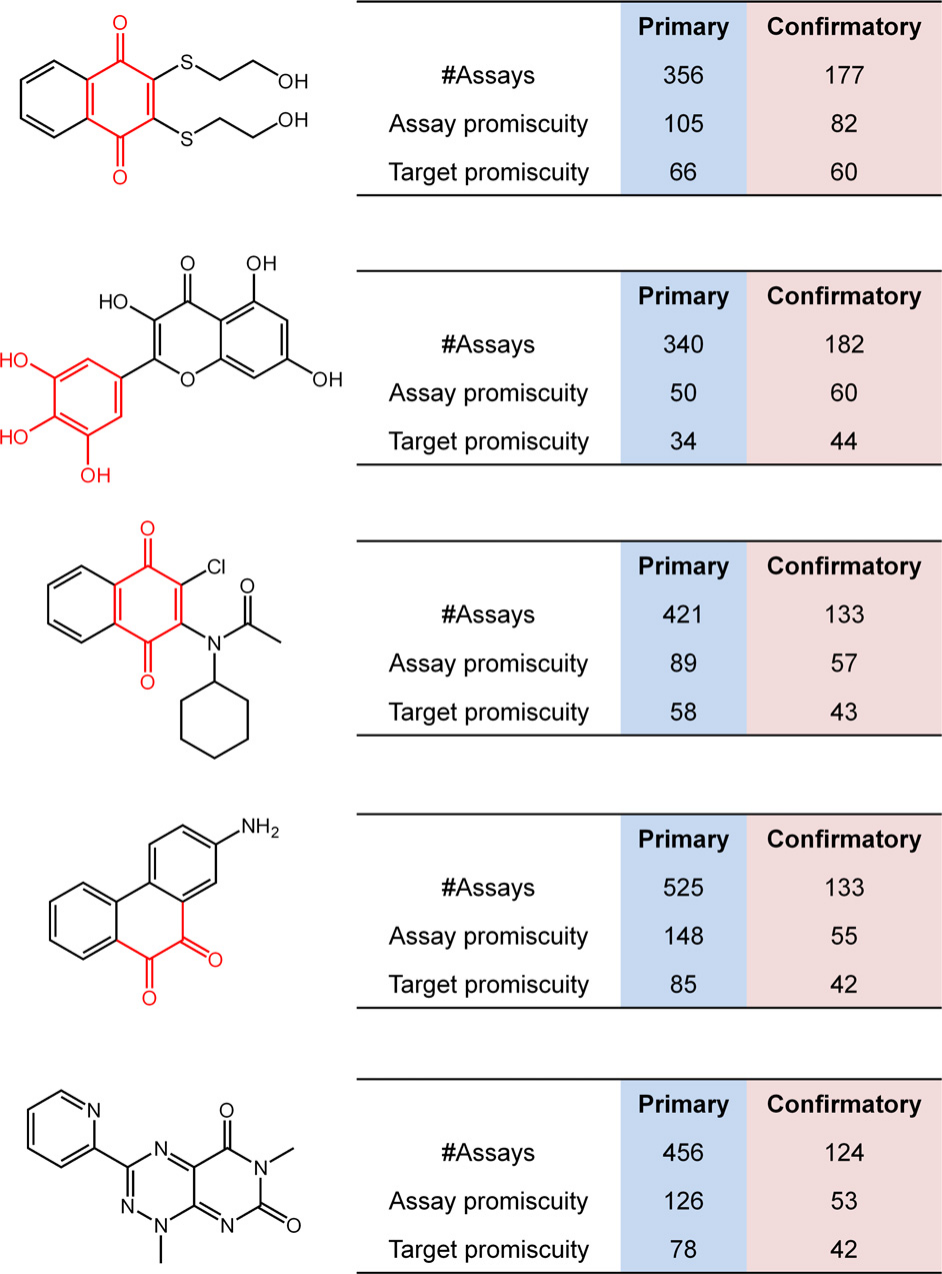
Highly promiscuous compounds. Shown are five exemplary highly promiscuous compounds. For each compound, the number of assays it was tested in and its assay and target promiscuity are reported. Four of these five compounds contain PAINS substructures (red).

Fig 7 shows examples of highly promiscuous compounds that were active in more than 100 or 200 assays and largely responsible for increases in the average over median degree of promiscuity. Most of these compounds contained PAINS substructures [7,8] and were thus prone to assay artifacts. The filter for PAINS substructures in compounds was implemented using pattern checker [25] available in ZINC 15 in which a list of 480 SMARTS patterns was provided [26]. It should be noted that different implementations of PAINS might result in different mappings due to the conversion of original structural representations into SMARTS or the generation of different SMARTS variants [27]. In addition, different sets of fragments might be used or substructure search routines.

Taken together, the results revealed that assay promiscuity was higher than target promiscuity, as we would anticipate. However, the differences were small, as the average degree of assay promiscuity only increased by 1.3 and 0.4 in primary and confirmatory assays, respectively. The differences were even smaller for median promiscuity degrees. In addition, the mean and median degrees of assay or target promiscuity also only differed by less than 1 or 2.

## Discussion

Target promiscuity of drugs and other bioactive compounds has thus far been studied on the basis of available activity annotations. Most recent surveys exclusively considering high-confidence activity data have resulted in average degrees of target promiscuity of 5.9 for approved drugs and 1.5 for bioactive compounds from medicinal chemistry sources [13]. Furthermore, the average degree of target promiscuity of compounds taken from confirmatory bioassays was 2.5 and thus also small [14]. Promiscuity estimates were generally higher for drugs than bioactive compounds. The higher degree of promiscuity among drugs might result from more extensive testing, but this remains uncertain. It is also possible that drug candidates that are successful in clinical trials might be more promiscuous than others.

Promiscuity analyses reported so far were based on known activity annotations, without taking assay frequencies or inactivity records into account, which are not available in major compound databases. This has generally been a point of concern, although very large volumes of activity data are already accessible, from which statistically meaningful trends can likely be derived. In light of data incompleteness or sparseness, it is frequently assumed that mining of compound activity annotations inevitably underestimates true compound promiscuity. This is likely the case although it remains unclear how large deviations from current promiscuity estimates might be.

We have set out to address these issues and further refine promiscuity analysis. Since it will hardly be possible to obtain a complete, or nearly complete, compound-target activity matrix any time soon, if at all, promiscuity analysis can at present only be further extended through incorporation of screening data. In addition, to address data sparseness concerns, compounds must be identified that have been extensively tested against many different targets.

Therefore, we have carried out a large-scale promiscuity analysis focusing on extensively assayed compounds. To our knowledge, this type of analysis is unprecedented. As a basis of our study, assay data were taken from PubChem and assay frequencies determined for all available compounds, which required substantial data curation efforts. For the first time, we also used primary screening data in promiscuity analysis to identify most extensively tested compounds. Because activity annotations from primary screening assays were only approximate in nature, multiple assays were frequently available for the same target, and a limited amount of cell-based assays was also considered, assay promiscuity was distinguished from target promiscuity and separately analyzed.

A subset of ~437,000 compounds was identified that were extensively tested in hundreds of assays against hundreds of targets. These compounds were subjected to promiscuity analysis in which primary and confirmatory assay data were separately considered. As expected, we found that assay promiscuity was generally higher than target promiscuity. However, the differences were surprisingly small, only on the order of 1, as reported above.

Given that primary screening data and extensively assayed compounds were used in our analysis, it was anticipated to observe higher degrees of target promiscuity for active compounds than previously reported. Average degrees of target promiscuity of 3.4 and 2.6 were determined for primary and confirmatory assays, respectively. These promiscuity degrees were only moderately higher, even for primary screening assays, than previously determined for ChEMBL compounds with available high-confidence activity data. We also detected small subsets of highly promiscuous screening hits, which led to an increase in average target promiscuity over median promiscuity. Highly promiscuous compounds often contained PAINS substructures and were thus likely to cause assay artifacts. Accordingly, median values might better estimate promiscuity degrees, at least for compounds from screening sources. The median degree of target promiscuity was 2.0 for both primary and confirmatory assays and thus only slightly higher than the corresponding value of 1.5 for ChEMBL compounds.

In conclusion, as revealed by our analysis, target promiscuity remained at a low level for bioactive compounds, even when studying the most extensively assayed compounds that are currently available. These findings lend further support to previously drawn conclusions that bioactive compounds are in general only moderately promiscuous and less promiscuous than drugs. One possible explanation would be that drugs are much more intensively investigated and tested for additional targets than bioactive compounds, for example, in many drug repurposing projects. Alternatively, given that drugs originate from the pool of bioactive compounds, these results also support the idea of a “promiscuity enrichment model”. The underlying hypothesis is that promiscuous compounds are preferentially selected for therapeutic efficacy during clinical evaluation and ultimately become drugs. This requires, however, that desired therapeutic effects due to substantial promiscuity outweigh unwanted side effects that are also possible.

## References

[1] Paolini GV, Shapland RH, van Hoorn WP, Mason JS, Hopkins AL. Global mapping of pharmacological space. Nat. Biotechnol. 2006;24: 805–815. 16841068 10.1038/nbt1228

[2] Boran AD, Iyengar R. Systems approaches to polypharmacology and drug discovery. Curr. Opin. Drug Discov. Devel. 2010;13: 297–309. 20443163 PMC3068535

[3] Jalencas X, Mestres J. On the origins of drug polypharmacology. Med. Chem. Comm. 2013;4: 80–87.

[4] Knight ZA, Lin H, Shokat KM. Targeting the cancer kinome through polypharmacology. Nat. Rev. Cancer 2010;10: 130–137. 10.1038/nrc2787 20094047 PMC2880454

[5] Hu Y, Bajorath J. Compound promiscuity—what can we learn from current data. Drug Discov. Today 2013;18: 644–650. 23524195 10.1016/j.drudis.2013.03.002

[6] Lu JJ, Pan W, Hu YJ, Wang YT. Multi-target drugs: the trend of drug research and development. PLoS ONE 2012;7: e40262 10.1371/journal.pone.0040262 22768266 PMC3386979

[7] Baell JB, Holloway GA. New substructure filters for removal of pan assay interference compounds (PAINS) from screening libraries and for their exclusion in bioassays. J. Med. Chem. 2010;53: 2719–2740. 10.1021/jm901137j 20131845

[8] Baell JB, Walters MA. Chemical con artists foil drug discovery. Nature 2014;513: 481–483. 10.1038/513481a 25254460

[9] Law V, Knox C, Djoumbou Y, Jewison T, Guo AC, Liu Y, et al DrugBank 4.0: shedding new light on drug metabolism. Nucleic Acids Res. 2014;42: D1091–1097. 10.1093/nar/gkt1068 24203711 PMC3965102

[10] Gaulton A, Bellis LJ, Bento AP, Chambers J, Davies M, Hersey A, et al ChEMBL: a large-scale bioactivity database for drug discovery. Nucleic Acids Res. 2011;40: D1100–D1107. 10.1093/nar/gkr777 21948594 PMC3245175

[11] Bento AP, Gaulton A, Hersey A, Bellis LJ, Chambers J, Davies M, et al The ChEMBL bioactivity database: an update. Nucleic Acids Res. 2014;42: D1083–D1090. 10.1093/nar/gkt1031 24214965 PMC3965067

[12] Wang Y, Xiao J, Suzek TO, Zhang J, Wang J, Zhou Z, et al PubChem’s BioAssay database. Nucleic Acids Res. 2012;40: D400–D412. 10.1093/nar/gkr1132 22140110 PMC3245056

[13] Hu Y, Bajorath J. High-resolution view of compound promiscuity [v2; ref status: indexed, http://f1000r.es/1ig]. F1000Res. 2013; 2: 144. 10.12688/f1000research.2-144.v1 PMC3799544 24358872

[14] Hu Y, Bajorath J. What is the likelihood of an active compound to be promiscuous? systematic assessment of compound promiscuity on the basis of PubChem confirmatory bioassay data. AAPS J. 2013;15: 808–815. 10.1208/s12248-013-9488-0 23605807 PMC3691425

[15] Hu Y, Jasial S, Bajorath J. Promiscuity progression of bioactive compounds over time. [v2; ref status: indexed, http://f1000r.es/5h4] F1000Res. 2015;4: 118. 10.12688/f1000research.6473.1 PMC4448747 26064479

[16] Hu Y, Bajorath J. Monitoring drug promiscuity over time [v2; ref status: indexed, http://f1000r.es/4oa] F1000Res. 2014;3: 218. 10.12688/f1000research.5250.1 PMC4207249 25352982

[17] Lusher SJ, McGuire R, van Schaik RC, Nicholson CD, de Vlieg J. Data-driven medicinal chemistry in the era of big data. Drug Discovery Today 2014;19: 859–868. 10.1016/j.drudis.2013.12.004 24361338

[18] Hu Y, Bajorath J. Learning from 'big data': compounds and targets. Drug Discovery Today 2014;19: 357–360. 10.1016/j.drudis.2014.02.004 24561327

[19] Mestres J, Gregori-Puigjane E, Valverde S, Sole RV. Data completeness–the achilles heel of drug-target networks. Nat. Biotechnol. 2008;26: 983–984. 10.1038/nbt0908-983 18779805

[20] Jacoby E. Chemogenomics: drug discovery’s panacea? Mol. BioSyst. 2006;2: 218–220. 16880939 10.1039/b603004c

[21] Canny SA, Cruz Y, Southern MR, Griffin PR. PubChem promiscuity: a web resource for gathering compound promiscuity data from PubChem. Bioinformatics 2012;28: 140–141. 10.1093/bioinformatics/btr622 22084255 PMC3276228

[22] Available: https://www.ncbi.nlm.nih.gov/pcassay/limits.

[23] Jasial S, Hu Y, Bajorath J. PubChem compounds tested in primary and confirmatory assays. ZENODO 2016; 10.5281/zenodo.44593

[24] Wassermann AM, Lounkine E, Hoepfner D, Le Goff G, King FJ, Studer C, et al Dark chemical matter as a promising starting point for drug lead discovery. Nat. Chem. Biol. 2015;11: 958–966. 10.1038/nchembio.1936 26479441

[25] Available: http://zinc15.docking.org/patterns/home/.

[26] Sterling T, Irwin JJ. ZINC 15—ligand discovery for everyone. J. Chem. Inf. Model. 2015;55: 2324–2337. 10.1021/acs.jcim.5b00559 26479676 PMC4658288

[27] Baell JB. Screening-based translation of public research encounters painful problems. ACS Med. Chem. Lett. 2015;6: 229–234. 25941544 10.1021/acsmedchemlett.5b00032 PMC4416424

